# Supplementary figures and images for: Genetic analysis reveals an east-west divide within North American Vitis species that mirrors their resistance to Pierce’s disease
Source: PLoS One. 2020 Dec 18;15(12):e0243445. doi: 10.1371/journal.pone.0243445 (PMC7748146; doi:10.1371/journal.pone.0243445)

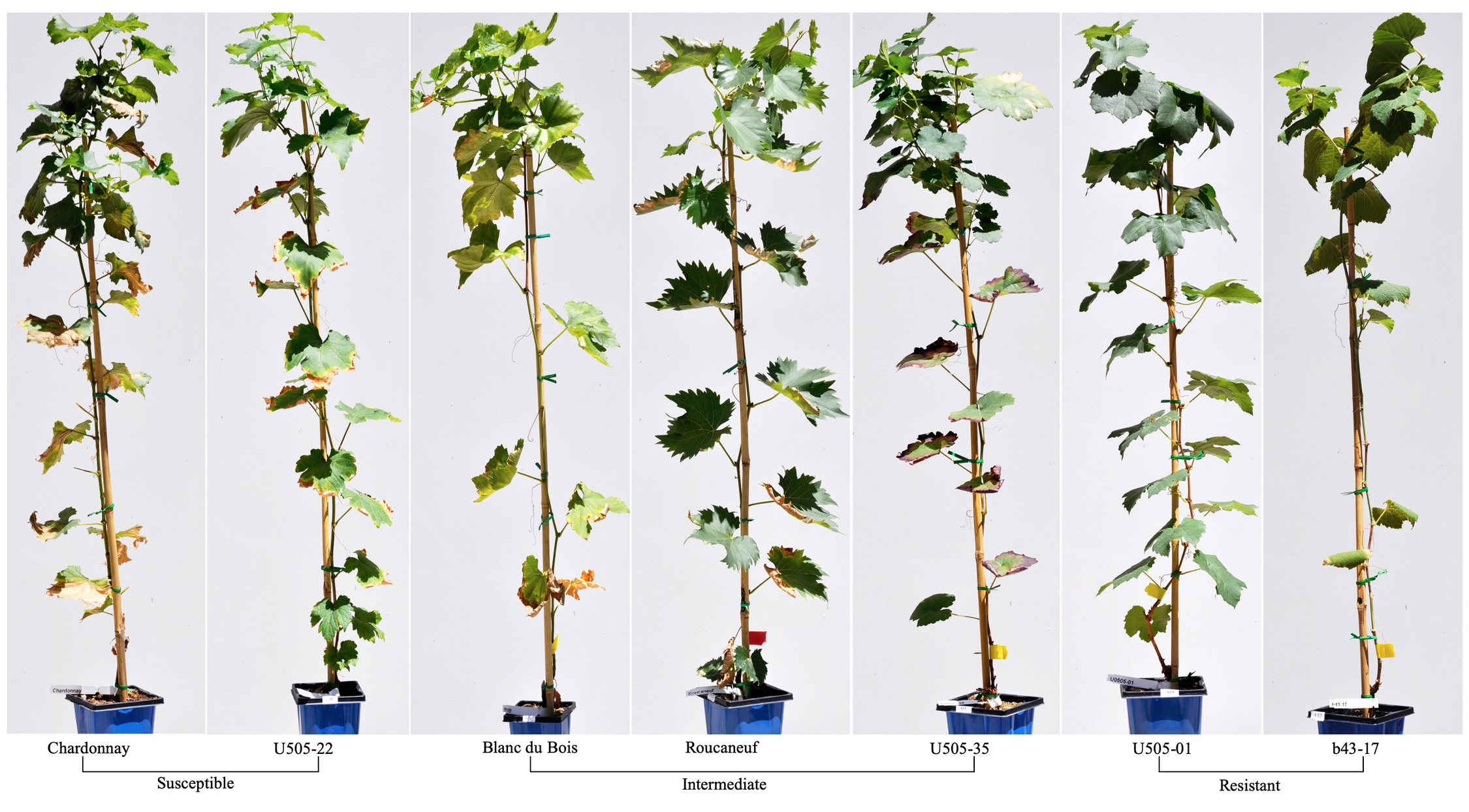

Supplement: S1 Fig — The reference plants were included in all greenhouse screening experiments to compare screen results across different experiments and years. (TIF) [file pone.0243445.s001.tif]

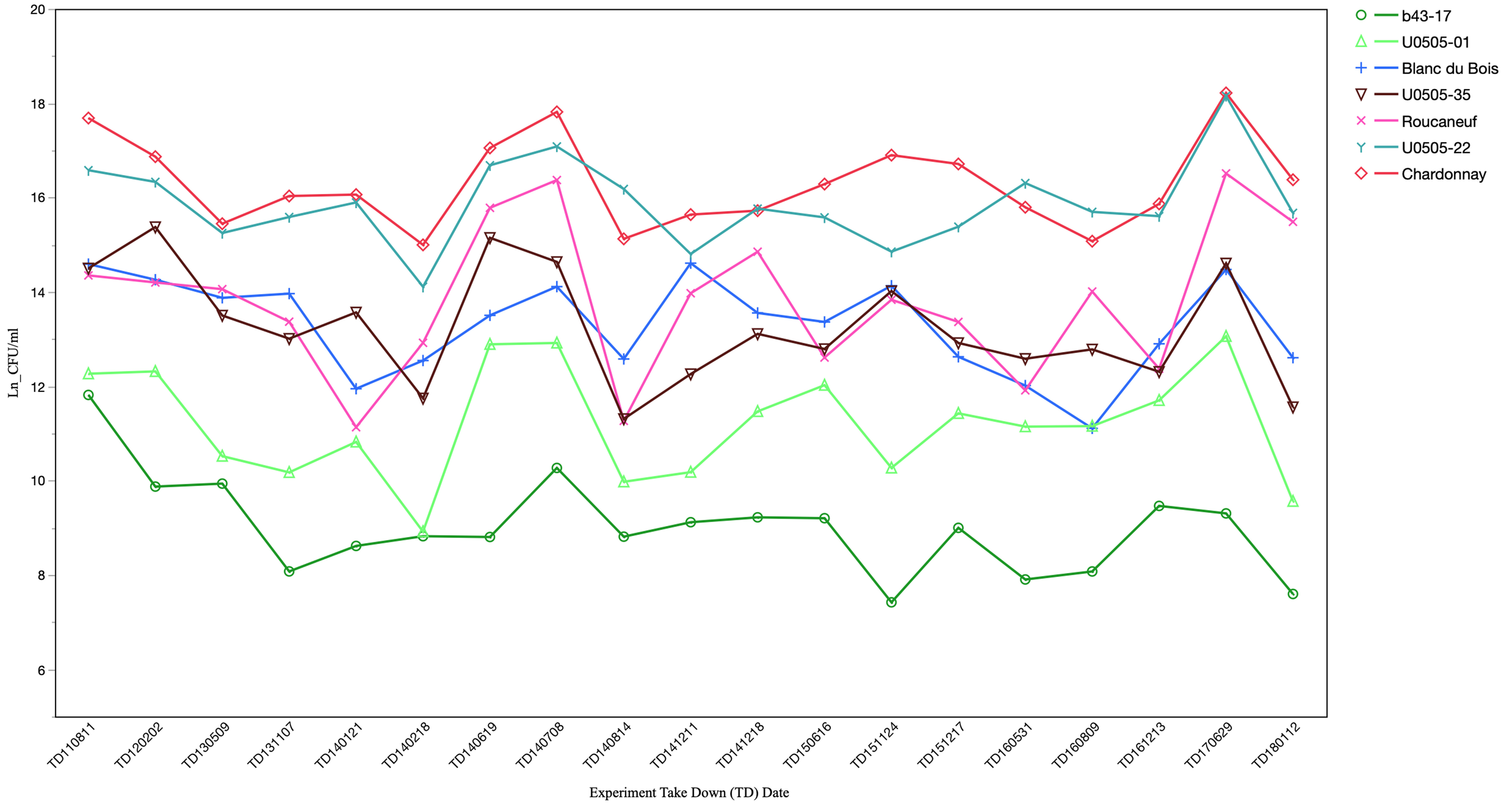

Supplement: S2 Fig — (TIF) [file pone.0243445.s002.tif]

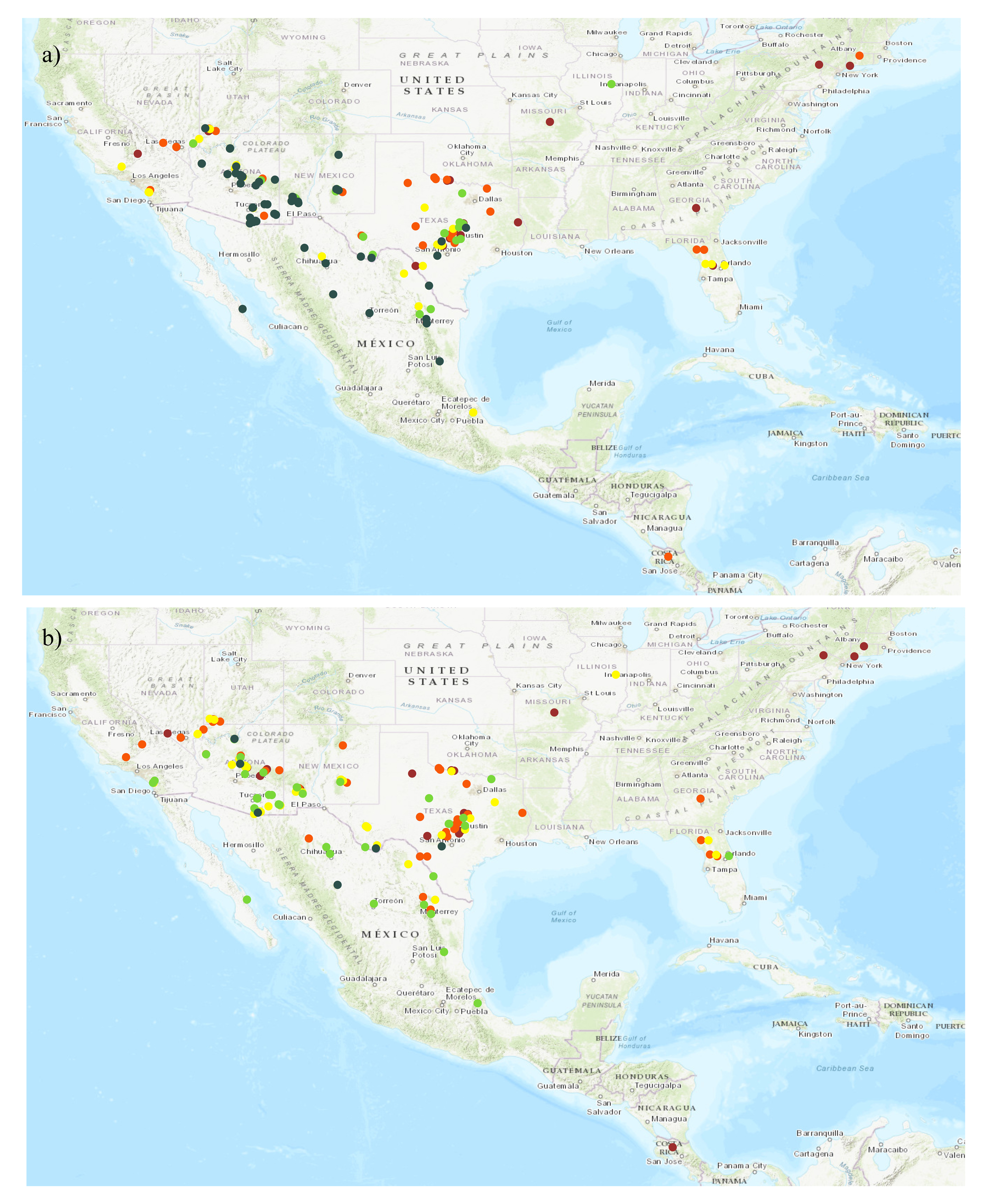

Supplement: S3 Fig — a) Cane maturation index; b) Leaf scorch/leaf loss. For both a and b, five categories were used: 1 (0–1), 2 (1.1–2.0), 3 (2.1–3.0), 4 (3.1–4), and 5 (4.1and above). The color scheme employed in Fig 3 was used for the five categories (dark green = 1, light green = 2, yellow = 3, orange = 4, and burgundy = 5). (TIF) [file pone.0243445.s003.tif]

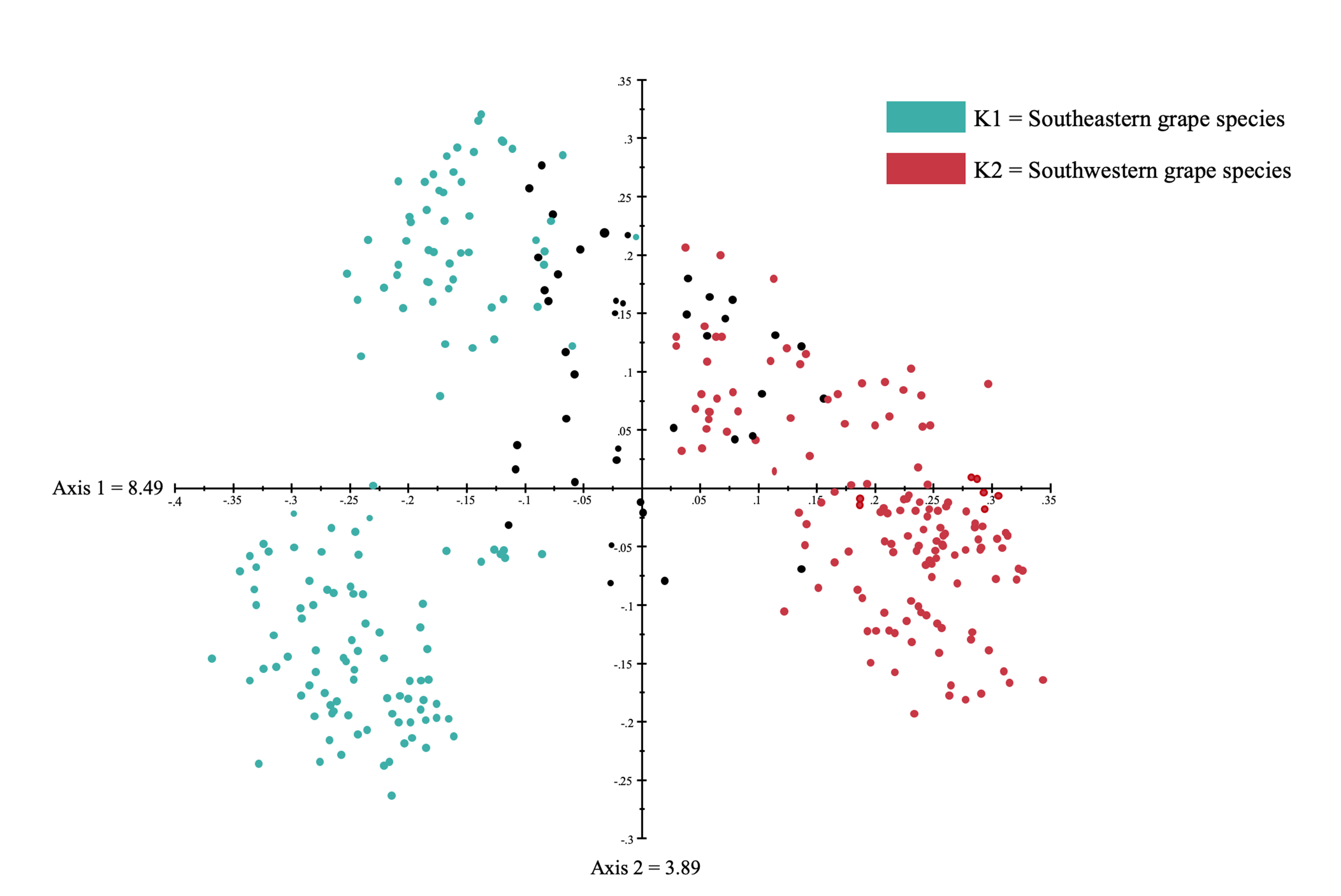

Supplement: S4 Fig — Axis 1 and 2 represent 8.49 and 3.89 percent of the variation, respectively. The color coding of STRUCTURE assignment was used for the PCoA display (bright cyan = group 1, moderate red = group 2, and black = admixture). (TIF) [file pone.0243445.s004.tif]
