# Supplementary material for: Genetic analysis reveals an east-west divide within North American Vitis species that mirrors their resistance to Pierce’s disease
Source: PLoS One. 2020 Dec 18;15(12):e0243445. doi: 10.1371/journal.pone.0243445 (PMC7748146; doi:10.1371/journal.pone.0243445)
Supplement: Attachment — Submitted filename: Response to reviewers.docx [file pone.0243445.s012.docx]

PLOS ONE

PONE-D-20-26579
Genetic analysis reveals an east-west divide within North American *Vitis* species that mirrors their resistance to Pierce's disease.

Response to Editor and Reviewer Comments

1. Please ensure that your manuscript meets PLOS ONE's style requirements, including those for file naming.

Yes, the manuscript meets PLOS One’s style requirements.

2. We note that Figures 1, 2 and S3 in your submission contain map images which may be copyrighted. All PLOS content is published under the Creative Commons Attribution License (CC BY 4.0), which means that the manuscript, images, and Supporting Information files will be freely available online, and any third party is permitted to access, download, copy, distribute, and use these materials in any way, even commercially, with proper attribution. For these reasons, we cannot publish previously copyrighted maps or satellite images created using proprietary data, such as Google software (Google Maps, Street View, and Earth). For more information, see our copyright guidelines: <http://journals.plos.org/plosone/s/licenses-and-copyright>.

 We require you to either (1) present written permission from the copyright holder to publish these figures specifically under the CC BY 4.0 license, or (2) remove the figures from your submission:

2.1.    You may seek permission from the original copyright holder of Figures 1, 2 and S3 to publish the content specifically under the CC BY 4.0 license.

 We recommend that you contact the original copyright holder with the Content Permission Form (<http://journals.plos.org/plosone/s/file?id=7c09/content-permission-form.pdf>) and the following text:

“I request permission for the open-access journal PLOS ONE to publish XXX under the Creative Commons Attribution License (CCAL) CC BY 4.0 (<http://creativecommons.org/licenses/by/4.0/>). Please be aware that this license allows unrestricted use and distribution, even commercially, by third parties. Please reply and provide explicit written permission to publish XXX under a CC BY license and complete the attached form.”

 Please upload the completed Content Permission Form or other proof of granted permissions as an "Other" file with your submission.

 In the figure caption of the copyrighted figure, please include the following text: “Reprinted from [ref] under a CC BY license, with permission from [name of publisher], original copyright [original copyright year].”

 2.2.    If you are unable to obtain permission from the original copyright holder to publish these figures under the CC BY 4.0 license or if the copyright holder’s requirements are incompatible with the CC BY 4.0 license, please either i) remove the figure or ii) supply a replacement figure that complies with the CC BY 4.0 license. Please check copyright information on all replacement figures and update the figure caption with source information. If applicable, please specify in the figure caption text when a figure is similar but not identical to the original image and is therefore for illustrative purposes only.The following resources for replacing copyrighted map figures may be helpful:

 USGS National Map Viewer (public domain): <http://viewer.nationalmap.gov/viewer/>The Gateway to Astronaut Photography of Earth (public domain): <http://eol.jsc.nasa.gov/sseop/clickmap/>Maps at the CIA (public domain): <https://www.cia.gov/library/publications/the-world-factbook/index.html> and <https://www.cia.gov/library/publications/cia-maps-publications/index.html>NASAEarth Observatory (public domain): <http://earthobservatory.nasa.gov/>Landsat: <http://landsat.visibleearth.nasa.gov/>

USGS EROS (Earth Resources Observatory and Science (EROS) Center) (public domain): [http://eros.usgs.gov/#](http://eros.usgs.gov/)Natural Earth (public domain): <http://www.naturalearthdata.com/>

The three maps in question were made with ArcGIS software and we cite this software with complete reference information and a link in the manuscript. See line 228 - 233.

“All maps presenting STRUCTURE assignment, and PD evaluation results were created using ArcGIS® software by Esri (ArcGIS® and ArcMap™, [www.esri.com](http://www.esri.com/)) [46”.

In the revised version, we added the source information for the base map to further facilitate the reader’s understanding of how these maps were created.

3. Please upload a copy of Supporting Information Tables 1-7 which you refer to in your text.

The Titles of the Supporting Information is included after the references. The seven tables are in one file titled “Supplemental Tables 1 through 7”

**Reviewer #1:**

This study used an extensive sampling of Vitis species and populations from the whole North America continent to study the phylogenetic relationships of grape germplasm and their correlation with pierce’s disease resistance. This study paid much attention and works to evaluate disease resistance by quantifying the level of bacteria in stems and measuring Pierce’s disease symptoms on the canes and leaves. Results of 20 SSR markers to genotype 326 accessions of grape species well suggested a geographical divide in the southwest and southeastern US and Mexico. Two hundred sixty-six of these accessions, and an additional 12 PD resistant hybrid cultivars developed from southeastern US grape species, were evaluated for PD resistance. This work provide clear phylogenetic pattern for the grape genus in North America with interesting pierce’s disease resistance evaluation.

But regretfully, no analyses were performed to test the correlation between this disease resistance trait and their phylogenetic and biogeographic relationships or explanation for the disease resistance pattern in North American grapes. Also, Results are too long and Discussion looks a little thin. Anyway, this study revealed an interesting phylogenetic pattern and the PD resistance pattern in North American grapes, and it is worthy to be accepted by the journal with some improvements

We carried out a correlation analysis for the three traits and the results are presented in lines 264-266. “The correlation analysis indicates that overall ELISA results had a 75% and 68% correlation to CMI and LS/LL values, respectively, and CMI results had a 67% correlation with LS/LL.”

Furthermore, Table 4a and 4b present the results for comparison of the three traits between the two main geographic groups. We also provide a possible explanation in the Discussion, see lines 521-527.

“A possible explanation for this disparity is that the southeastern US grape species are not resistant to PD, but instead are tolerant and the stem and leaf desiccation symptoms of PD are suppressed by wetter, more humid conditions. After infection, *X. fastidiosa* inhabits and spreads within the xylem. The infection initiates a plant response that results in vascular occlusions, predominantly tyloses, to limit bacterial spread. This response also decreases water transport up to 90% in susceptible plants [53]. Warm humid climates promote more vegetative growth, which may allow infected plants to dilute the infection and tolerate blocked vessels for a longer time”.

1. Phylogenetic history within Vitis is an interesting puzzle and topological relationships for the whole Vitis group including North America have been studied many times before. All these works should be compared and discussed in this study. Further, it is better to present a topological tree to show their phylogenetic relationships.

The objective of this study was to examine the distribution of PD resistance within the North American grape species and gain a better understanding of PD resistance within the Eastern US and Western US grape species. Furthermore, we don’t have a complete representation of all the grape species in this study and results would not provide a complete picture on the topological relationships.

2. PD resistance data seems not to be well match phylogenetic divide of North American grapes, there is no clear east-west divide as shown in phylogenetic data. A correlation analyses should be used. Climate is a possible explanation for their different disease resistance, but there must have many other causes, such as from an evolutionary view.

Please see comments above. We do not have an appropriate collection of Vitis species to examine evolutionary relationships, nor was it our intent.

3. Sample information including locations or sources for all the 346 accessions should be given in table or as a supplement.

Supplemental tables are provided with all this information.

4. To have a better presentations, subgroups in the two main groups should be also marked in Figure 2. If possible, hybridization zone and the Sierra Madre Oriental should also be noted on Figure 2 since they have been mentioned many times in the discussion.

We feel that Figure 2 already contains too much information and that adding more information would make it too complex.

5. Line 52, Muscadinia rotundifolia --> Vitis rotundifolia

We have made this change, although the use of *Muscadinia* as a subgeneric taxon (if not generic) is clearly justified given the multitude of differences that distinguish *Vitis* species from *Muscadinia* on the basis of genetic, cytogenetic, morphological and pest and disease resistance traits. This is an old argument in the grape breeding/genetic community and we feel it is important to keep it alive as newer members of this community become active.

We have added the following sentence in Lines 56 - 59 This taxon also possesses a broad array of genetic and morphological differences from all other *Vitis* species and creates sterile hybrids with them, leading to its inclusion in a separate subgenus or genus – *Muscadinia*.
